# Combined Intramedullary Nailing and Interfragmentary Screws in Distal Tibial Fractures With Articular Extension

**DOI:** 10.7759/cureus.87033

**Published:** 2025-06-30

**Authors:** Imad Marzak, Abdullah Zaher, Jaouad Yasser, Noureddine Sekkach

**Affiliations:** 1 Orthopedic Surgery, Cheikh Zaid International University Hospital, Rabat, MAR; 2 Orthopedics and Traumatology, Delafontaine Hospital, Saint-Denis, FRA; 3 Orthopedic Surgery, Delafontaine Hospital, Saint-Denis, FRA

**Keywords:** american orthopedic foot and ankle society (aofas) ankle/hindfoot score, ankle fractures, distal tibial fractures (dtf), inrtra-articular fracture, intramedullary nails

## Abstract

The treatment of distal tibial fractures with an articular fracture line remains controversial. Intramedullary nailing allows for the maintenance of good frontal and sagittal alignment, as well as rapid and satisfactory functional recovery. In this retrospective case series, we present our department's experience with 20 patients presenting with AO 43C1 and 43C2 fractures, treated with intramedullary nailing combined with interfragmentary screw fixation over an eight-year period, with a follow-up of 18 months. Postoperative alignment was assessed using radiographic measurements of the anterior distal tibial angle (ADTA) and lateral distal tibial angle (LDTA), immediately and at follow-up. Functional outcomes were evaluated using the American Orthopaedic Foot and Ankle Society (AOFAS) score. Descriptive statistical analysis was used to report means, standard deviations, and proportions. Acceptable alignment was achieved in 95% of patients. The AOFAS score exceeded 86% in 88% of patients, indicating excellent outcomes. Interfragmentary screw fixation prior to nailing improved construct stability, minimized malalignment risk, and enhanced functional recovery.

## Introduction

The management of distal tibial metaphyseal fractures aims to ensure stable osteosynthesis while minimizing soft tissue damage. These fractures, particularly when involving intra-articular extension, pose specific challenges due to their anatomical complexity. This study aims to evaluate the outcomes of intramedullary nailing combined with interfragmentary screw fixation in patients with simple intra-articular distal tibial fractures (AO 43C1 and 43C2). Our hypothesis is that this combined approach improves both alignment and functional recovery.

## Materials and methods

Study design and setting

**Figure 1:**
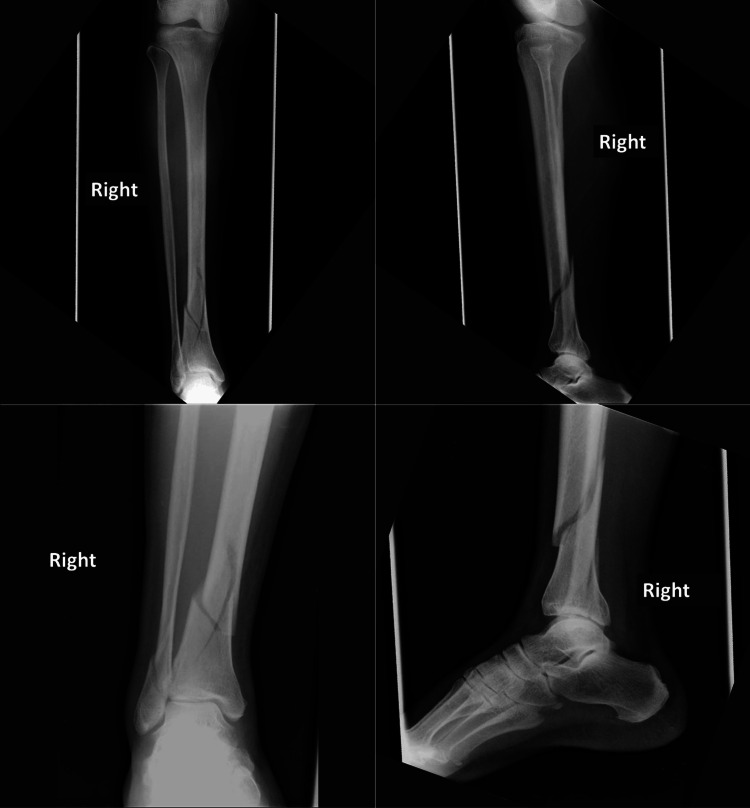
A 43-year-old female patient presented following a road traffic accident with trauma to the right leg and ankle. Anteroposterior and lateral radiographs of the leg revealed a distal quarter tibial fracture with intra-articular extension associated with a fracture of the lateral malleolus. Image credit: Authors

We conducted a retrospective case series based on anonymized radiographic data collected as part of routine clinical care. Radiographs were obtained from patients presenting to the Orthopedic Department of the Saint-Denis Hospital Center between 2015 and 2023 (Figure 1). We included patients with distal tibial fractures featuring an intra-articular extension, treated with intramedullary nailing and an interfragmentary compression screw.

Patients

A total of 374 patient records involving tibial fractures treated surgically with intramedullary nailing were initially reviewed. Of these, 354 cases were excluded due to one or more of the following criteria: follow-up period of less than 18 months, absence of an intra-articular fracture line, or fractures involving the distal tibial epiphysis. The final study population included 20 adult patients presenting with a recent fracture of the distal quarter of the tibia, characterized by a simple intra-articular split (classified as AO 43C1 or 43C2), whether displaced or non-displaced. All patients were treated with intramedullary nailing supplemented by interfragmentary screw fixation and had a minimum follow-up of 18 months.

Surgical method

All patients underwent surgery under regional anesthesia, without the use of a pneumatic tourniquet. They were positioned supine, with the affected leg hanging and supported by a knee bar. Knee flexion had to reach at least 90° to allow proper insertion of the nail.

The first step involved reducing and fixing the intra-articular fracture line via percutaneous screw fixation under fluoroscopic guidance (one or two screws placed perpendicular to the fracture line). This step helps prevent "blowout" during nail insertion and avoids secondary malalignment of the distal tibia in conjunction with distal locking screws.

A transpatellar tendon approach was then performed. After initial bone penetration with a curved "pigtail" awl and the insertion of a ball-tipped guidewire into the medullary canal, progressive reaming was performed via a slow-speed motor, enlarging the canal to a diameter 1.5 mm greater than that of the planned nail.

**Figure 2:**
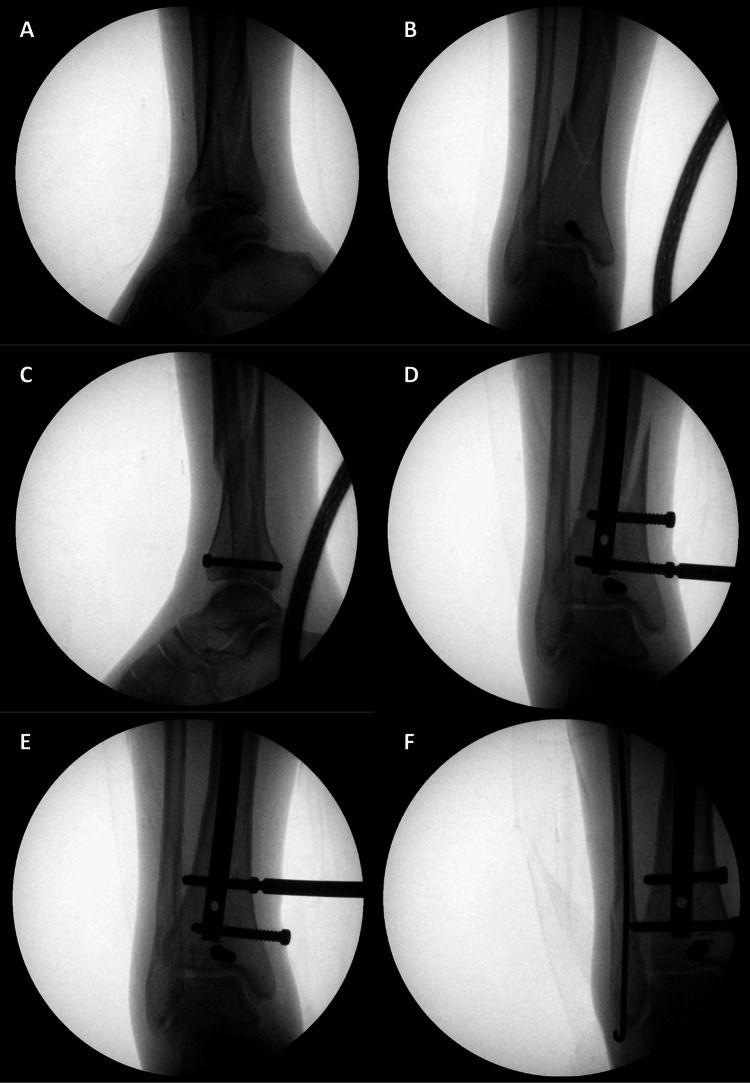
Fluoroscopic images of the osteosynthesis steps for a distal tibial fracture with articular extension. (A) Lateral fluoroscopic image showing the intra-articular fracture line; (B, C) interfragmentary screw fixation; (D, E) locking screws used for interfragmentary compression; (F) fibular fracture fixation via a Rush pin. Image credit: Authors

Proximal locking was achieved via the dedicated targeting guide with automatic locking. Distal locking was typically performed freehand under fluoroscopic control after removing the knee bar and verifying the absence of rotational malalignment (Figure 2).

In some patients, distal locking also allows compression of the intra-articular fracture line in combination with the additional screws. It is recommended to slightly loosen the supplementary screws before tightening the distal locking screws to avoid valgus displacement of the distal tibial epiphysis.

Method of outcome evaluation

At the final follow-up (>18 months), two quantitative and eight qualitative variables were assessed. The lateral distal tibial angle (LDTA) and anterior distal tibial angle (ADTA) were measured using standard radiographic techniques described in the literature [1]. The LDTA is defined as the angle between the anatomical axis of the tibia and the distal tibial articular surface in the coronal plane. The ADTA is defined as the angle between the mechanical axis of the tibia and the ankle joint orientation line in the sagittal plane (normal range: 80° ± 3°) [2,3]. These measurements were performed on weight-bearing anteroposterior and lateral radiographs, following the method detailed by Carrara et al. [1].

The functional outcomes were assessed via qualitative variables derived from the American Orthopedic Foot and Ankle Society (AOFAS) score [4]. The reliability and validity of this score have been well documented. It evaluates pain, function, activity level, use of walking aids, walking distance, limp, limitation of hindfoot range of motion, and sagittal alignment. Each parameter contributes to a total score ranging from 0 to 100 points, with higher scores indicating better functional outcomes.

## Results

Patient characteristics

The mean age of the patients was 45 years (Table 1). Road traffic accidents were the most common mechanism of injury, accounting for 60% of cases, followed by domestic accidents (15%), work-related injuries (10%), sports accidents (5%), and crushing injuries (5%). The majority of fractures were closed (90%), with only 10% being classified as open fractures (Gustilo type I). In 75% of cases, the tibial fracture was associated with a concomitant fibular fracture, which was treated simultaneously. Regarding osteosynthesis, one distal locking screw was used in 45% of patients, while two screws were used in 55% of cases to achieve compression and stabilization of the intra-articular fracture line.

**Table 1:** Patient characteristics.

Parameter	Value
Mean age	45 years
Sex	-
Male	75% (n = 15)
Female	25% (n = 5)
Injury mechanism	-
Road traffic accident	60% (n = 12)
Domestic accident	15% (n = 3)
Work-related accident	10% (n = 2)
Sports injury	5% (n = 2)
Crushing injury	5% (n = 1)
Fracture type	-
Open (Gustilo type I)	10% (n = 2)
Closed	90% (n = 18)
History of diabetes	5% (n = 1)
Smoking	5% (n = 1)
Associated fibula fractures treated simultaneously	75% (n = 5)
Number of distal locking screws	-
1 screw	45% (n = 9)
2 screws	55% (n = 11)
Mean time to union	7.74 months
Delayed union	15% (n = 3)
Infection rate	0%
Symptomatic hardware	10% (n = 2)
Deep vein thrombosis	0%

Ankle alignment assessment

The mean degree of sagittal deformity was 1.20° (range: 0°-3°). The mean frontal deformity was 1.30° (range: 0°-3°). Acceptable alignment was achieved in 19 patients (95%). One patient presented with a 5° valgus deformity. Importantly, the final alignment, both in the frontal and sagittal planes, did not change after 18 months when compared with the immediate postoperative alignment.

Fracture healing

The mean time to consolidation in our series was 7.74 months (range: 3-26 months). However, three patients with delayed consolidation required dynamization of the nail within the first three months. Only one case of aseptic nonunion was observed in our series.

Functional outcomes

The functional AOFAS score was greater than 86% in 88% of the patients, indicating an excellent outcome (Table 2).

**Table 2:** Summary of the American Orthopaedic Foot and Ankle Society (AOFAS) score parameters.

Parameters	Mean ± Standard Deviation	Min – Max
Global AOFAS score (/100)	86.1 ± 11.8	40 – 95
Pain (/40)	38.0 ± 7.3	0 – 40
Function (/50)	38.4 ± 6.2	20 – 45
Activity limitations, support requirements	7.5 ± 2.2	0 – 10
Maximum walking distance, blocks	3.6 ± 1.5	0 – 5
Walking surfaces	3.7 ± 1.2	0 – 5
Gait abnormality	6.8 ± 2.4	0 – 8
Sagittal motion	6.4 ± 2.7	0 – 8
Hindfoot motion	4.0 ± 2.6	0 – 6
Stability and alignement (/10)	9.7 ± 1.0	5 – 10
AOFAS score ≥ 86%	88% (n = 18/20)	-

## Discussion

Distal tibial articular fractures represent less than 1% of all lower limb fractures and 3-10% of all tibial fractures [1]. The number assigned to distal tibia fractures in the AO classification is 43, with type B fractures including partial articular fractures and type C fractures encompassing complete articular fractures. Each type is further divided into subgroups on the basis of the degree of comminution. This study focused on fractures classified as 43C1 and 43C2.

These fractures are most often caused by high-energy trauma [2]. In our series, 85% of patients sustained high-energy trauma in various circumstances. Despite ongoing advancements in the surgical management of distal tibial fractures, the optimal surgical technique remains controversial. Plates, intramedullary nails, and external fixators are three effective surgical methods. No single method is universally appropriate for all types of distal tibial fractures [3].

Osteosynthesis of distal tibial fractures via percutaneous plates ensures stability and alignment while minimizing the risk of skin complications and infections [4,5]. Compared with those in the plate fixation group, Im et al. demonstratedshorter operative times with improved function in the intramedullary nailing group compared to the plate fixation group[6].

The challenges in reducing distal tibial fractures are often related to the flared morphology of the distal tibia, where the diameter is much larger than that of the nail. The distance between the nail and the tibial cortex does not allow for fracture reduction. To address this issue, several authors recommend the use of auxiliary locking screws to center the nail and achieve alignment and good reduction [7]. Fixation of distal tibial fractures via intramedullary nailing has been limited by the reduced bone-implant contact at the distal metaphysis. The flared cortical structure of the metaphysis allows a "wiping" effect of the distal fragment relative to the intramedullary nail, minimizing the intrinsic stability of the fixation compared with plate fixation. When the contact between the two fracture surfaces is minimal, a larger proportion of the mechanical load is borne by the nail and transmitted to the distal screws. This results in load-sharing between the distal screws and the metaphysis, which may lead to screw bending and failure of the construct. There are numerous techniques to increase the stability of osteosynthesis, including fibular fixation, multiplanar distal locking screws, and additional interfragmentary screws, prior to intramedullary nailing [8].

Fibular fractures are associated with 90% of cases, and approximately 15% of cases present significant injuries in the ipsilateral foot [9]. Fibular fixation is important for improving the outcomes of intramedullary nailing. Indeed, fibular fixation in the context of distal tibial fractures enhances the stability of the ankle joint and contributes to the reduction and healing of the tibial fracture [6].

For fractures with significant valgus angulation, fibular fixation can be used to facilitate reduction prior to intramedullary nailing. A biomechanical study confirmed that fibular fixation in conjunction with intramedullary nailing reduces the rates of nonunion and angular displacement [10]. Egol et al. [11] reported on 72 distal tibial and fibular fractures treated with intramedullary nailing, with or without fibular fixation. Patients treated with a fibular plate in addition to nailing had a lower incidence of late malalignment compared to those treated with nailing alone (4% vs. 13%, respectively) [12].

In the largest series of distal tibial fractures treated with locked intramedullary nailing, Ristiniemi et al. reported a union rate of 100% [12], but they reported deformities of varus and recurvatum of 10°. Their series included both extra-articular fractures and fractures with articular involvement. Complementary fixation of the articular fracture was performed after intramedullary nailing, whereas in our series, we opted to place interfragmentary screws for the osteosynthesis of the articular fracture before nailing, to avoid displacement of the articular fracture lines during reaming or nail insertion.

We had no cases of secondary displacement of the articular fragments after nailing. This has been reported by Nork et al. [13]in their study on intramedullary nailing of distal tibial fractures in 36 patients, 10 of whom had an articular fracture line [13]. Articular fracture fixation before nailing was advocated by Ehlinger et al. [14]and was proven in their series of 51 patients with distal tibial fractures, 13 of whom had articular fractures. Our results concerning alignment were satisfactory, which, as reported in the literature, is due to the use of fibular fixation, multiplanar distal locking screws, and additional interfragmentary screws. Ehlinger et al. [14] reported in their series two cases of axis deviation greater than 10° related to the absence of fibular fixation. Table 3 presents the main articles reporting cases or series of osteosynthesis associated with additional screw fixation in diaphyseal tibial fractures with an articular split.

**Table 3:** Main articles reporting cases or series of osteosynthesis associated with additional screw fixation in diaphyseal tibial fractures with an articular split.

Authors	Year	Number of patients	Frontal malalignment	Sagittal malalignment	Consolidation rate	Consolidation time	Functional score
Nork et al. [13]	2005	10	1 valgus (5°)	2 recurvatum (5°)	100%	23.5 weeks (13-57)	16.7 ± 15.6 (0.6-41.6 points)
Ehlinger et al. [14]	2010	13	-	-	97.6%	15.7 weeks (10-32)	87.8 points (40-100 points)
Mosheiff et al. [15]	1999	20	0	0	96%	15 weeks (12-24)	-

Early dynamization of the nail was performed prophylactically to achieve consolidation in four patients. We removed a proximal locking screw, which allowed proximal migration of the nail to ensure compression.

The functional outcome was assessed at a minimum of one year for 18 patients, and two patients were lost to follow-up. The functional AOFAS score was greater than 86% in 88% of the patients, indicating excellent results. The functional score in our series at the 18-month follow-up was greater than 86% in 88% of our patients, which is a very good result. A study published in the Journal of Bone and Joint Surgery (JBJS) [13] reported a similar outcome to ours at a two-year follow-up. 

The potential bias of this study lies in the accuracy of radiological measurements of the LDTA and ADTA angles. To address this, measurements were performed by two orthopedic surgeons, and a comparison of the results was made. Any measurements that differed were taken a second time.

## Conclusions

Simple articular fractures of the distal tibia treated with intramedullary nailing preceded by additional percutaneous interfragmentary screw fixation can achieve excellent rates of alignment and consolidation, with proper patient selection and appropriate surgical indications.
